# Random Access Preamble Design for 6G Satellite–Terrestrial Integrated Communication Systems

**DOI:** 10.3390/s25175602

**Published:** 2025-09-08

**Authors:** Min Hua, Zhongqiu Wu, Cong Zhang, Zeyang Xu, Xiaoming Liu, Wen Zhou

**Affiliations:** 1 The College of Information Science and Technology & Artificial Intelligence, Nanjing Forestry University, Nanjing 210037, China; 3230800451@njfu.edu.cn (Z.W.); cong-zhang@njfu.edu.cn (C.Z.); xzy152092@163.com (Z.X.); lxm@njfu.edu.cn (X.L.); 2 The School of Low-Altitude Equipment and Intelligent Control, Guangzhou Maritime University, Guangzhou 510725, China; wenzhou@ustc.edu

**Keywords:** satellite-terrestrial integrated communication systems (STICSs), 6G, Internet of Things (IoT), random access, a carrier frequency offset (CFO)-resistant preamble design, root set selection algorithm

## Abstract

Satellite–terrestrial integrated communication systems (STICSs) are envisioned to provide ubiquitous, seamless connectivity in next-generation (6G) wireless communication networks for massive-scale Internet of Things (IoT) deployments. This global coverage extends beyond densely populated areas to remote regions (e.g., polar zones, open oceans, deserts) and disaster-prone areas, supporting diverse IoT applications, including remote sensing, smart cities, intelligent agriculture/forestry, environmental monitoring, and emergency reporting. Random access signals, which constitute the initial transmission from access IoT devices to base station for unscheduled transmissions or network entry in terrestrial networks (TNs), encounter significant challenges in STICSs due to inherent satellite characteristics: wide coverage, large-scale access, substantial round-trip delay, and high carrier frequency offset (CFO). Consequently, conventional TN preamble designs based on Zadoff–Chu (ZC) sequences, as used in 4G LTE and 5G NR systems, are unsuitable for direct deployment in 6G STICSs. This paper first analyzes the challenges in adapting terrestrial designs to STICSs. It then proposes a CFO-resistant preamble design specifically tailored for STICSs and details its detection procedure. Furthermore, a dedicated root set selection algorithm for the proposed preambles is presented, generating an expanded pool of random access signals to meet the demands of increasing IoT device access. The developed analytical framework provides a foundation for performance analysis of random access signals in 6G STICSs.

## 1. Introduction

By 2029, the number of devices within the Internet of Things (IoT) networks is projected to reach 29 billion [1]. These devices range from low-cost environmental sensors to sophisticated instruments such as smartphones and robots [2]. They serve diverse applications critical to modern infrastructure and environmental stewardship, including remote sensing, smart cities, smart grids, intelligent transportation systems, agricultural and forestry monitoring, environmental surveillance and emergency reporting [1,3,4,5]. Seamless connectivity is paramount for facilitating data collection, exchange, and processing between these geographically dispersed IoT devices and users, thereby enabling intelligent decision-making systems across these domains [1,4,5,6]. Through the integration of sensing and communication (ISAC) capabilities, supported by technologies such as intelligent reflecting surfaces (IRS), along with the deep integration of artificial intelligence into the network architectures [7,8], the envisioned next-generation (6G) networks are expected to further amplify these demands. This progression will intensify the need for robust connectivity solutions to support massive and ubiquitous IoT deployments.

However, conventional terrestrial cellular communication networks, primarily designed for person-to-person communication in populated areas, struggle to provide ubiquitous coverage due to environmental and economic constraints. Consequently, regions with poor or unavailable terrestrial coverage, such as oceans, forests, polar areas, and remote sensing operation sites, remain underserved for IoT deployments [9,10,11,12,13]. Non-Terrestrial Networks (NTNs), comprising satellites and high-altitude platforms, are seen as a feasible solution to supplement terrestrial IoT networks and achieve ubiquitous coverage in 6G wireless communication systems. They are envisioned to provide essential communication backhaul for geographically dispersed IoT assets, particularly remote sensors and trackers [9,10,11,12]. The ongoing deployment of remote sensing constellations in China and globally further intensifies the demand for robust and scalable communication solutions for data transmission [14]. Significant satellite deployment efforts are underway, with the low Earth orbit (LEO) satellite market expected to grow to $12.51 billion by 2027 at a 24.2% compound annual growth rate (CAGR) [1]. Compared with geostationary Earth orbit (GEO) and medium Earth orbit (MEO) satellite communication systems, LEO satellite systems operate at a significantly lower orbital altitude. This results in substantially shorter propagation delays (typically 4–10 ms versus approximately 500 ms for GEO systems [15]), lower production and launch costs [16], and lower path loss. The decreased path loss contributes to an improved link budget relative to GEO or MEO systems, thereby reducing energy consumption for both devices in the uplink and satellite transmitters in the downlink. These characteristics make LEO constellations the preferred platform for satellite–terrestrial integrated communication systems (STICSs), as reflected in the broader literature [10,12,17], and constitute the main focus of this paper.

In terrestrial cellular systems, the random access (RA) procedure establishes uplink synchronization, identifies users, and resolves unscheduled data transmission. This process is initiated when an access device transmits an RA preamble on the physical random access channel (PRACH). Upon preamble detection, the base station identifies the preamble index within the assigned preamble pool and measures the round-trip propagation delay (sum of downlink and uplink delays). The preamble index is further utilized to confirm the unique identity of the detecting device, while the round-trip delay (RTD) adjusts subsequent uplink transmission timing on the physical uplink shared channel (PUSCH), ensuring time-aligned signals at the base station receiver. Current 4G Long Term Evolution (LTE) and 5G New Radio (NR) systems employ Zadoff–Chu (ZC) sequence-based CDMA preambles spread over dedicated PRACH resources in time and frequency [18,19].

However, adapting this TN preamble design to NTNs, particularly satellite communication systems, presents significant challenges due to unique propagation characteristics. The extended communication distance in satellite communication systems induces severe path loss, which degrades the received signal-to-noise ratio (SNR) and consequently impairs detection performance critical for IoT devices in remote scenarios. Furthermore, the RTD in satellite communication systems often exceeds the preamble duration used in TN, invalidating cyclic-shift-based device differentiation. Substantial Doppler shifts caused by rapid relative motion between the satellite and the access devices disrupt subcarrier orthogonality, leading to energy attenuation and leakage [20,21,22]. Additionally, the vast coverage areas of satellites necessitate support for a substantially larger pool of RA preambles than the terrestrial limit of 64 per PRACH resource defined in LTE/NR standards. This requirement is particularly critical to accommodate large-scale deployments of IoT devices accessing the network simultaneously over wide geographic areas.

To ensure compatibility with terrestrial RA signaling, extensive research has focused on designing preamble sequences for satellite communication systems, often leveraging the ZC sequence-based framework. Several studies [23,24,25,26] propose constructing long preamble sequences by concatenating multiple ZC sequences with different roots in the time domain. However, a larger subcarrier spacing (SCS) is required in [23,24] to help increase the system sensitivity to carrier frequency offset (CFO). Moreover, the significant propagation delay and path loss inherent in satellite channels are not fully addressed in [25,26]. Other designs concatenate a single-root ZC sequence with different cyclic shifts [27], but this also requires a larger SCS to ensure the maximum CFO does not exceed half the SCS for robustness. Furthermore, some studies integrate ZC sequences with other sequence types. For example, ref. [9] proposes a long preamble structure formed by cascading several short ZC sequences in the time domain, which are subsequently scrambled using a BPSK-modulated m-sequence. Similarly, ref. [28] introduces a novel PRACH format where part of repeated ZC sequences is scrambled using a BPSK-Golden sequence. Crucially, most of these studies [9,23,24,25,26,27] focus on designing only a single preamble. As highlighted in [28,29], the wide satellite coverage area and the increased scale of simultaneous RA attempts necessitate the availability of a significantly larger pool of preambles. Therefore, it is essential to develop a comprehensive preamble set comprising more preambles. In this context, analyzing the cross-correlation properties among these signals becomes crucial to ensure reliable detection and minimize multi-user interference.

Additionally, to simplify integrating 5G NR’s PRACH into NTN scenarios, several works assume that Global Navigation Satellite System (GNSS) functionality is embedded within each IoT device [9,12,30,31,32]. This enables devices to utilize GNSS-derived location and satellite ephemeris data for mobility management, compensating for Doppler effects and the large delays. The authors also previously proposed a pool of 128 random access preambles for satellite communication, assuming GNSS compensation for Doppler frequency shift and common RTD [32]. However, neglecting CFO is impractical. As indicated in [21,23], while the common Doppler shift and RTD relative to a reference point can be calculated and eliminated by the satellite, the device-specific RTD and, critically, device-specific CFO require specific treatment in NTN RA preamble design. These device-specific impairments may not be readily acquirable due to environmental factors or limitations in IoT device capability and configuration (e.g., oscillator instability). Therefore, in this paper, device-specific CFO and delay are incorporated into the preamble design, while common Doppler shift and RTD are assumed to be pre-compensated via GNSS. Accordingly, it is essential to develop a preamble pool that: (1) adheres to satellite communication requirements (e.g., wide coverage, large-scale access), (2) accounts for user-specific delay and frequency offsets, and (3) maintains compatibility with TN preamble designs.

Motivated by these critical requirements for enabling massive IoT connectivity, this paper introduces a pool of RA preamble signals optimized for LEO-based STICSs. Section 2 reviews the RA design in current TN and discusses the approach proposed in [32] for LEO-based STICSs. While the design in [32] satisfies several requirements, it fails to address device-specific CFO. Section 3 analyzes the impact of CFO on the approach presented in [32]. Section 4 proposes a CFO-resistant preamble design, upgraded from the design in [32], and details the corresponding detection procedure. Section 5 presents a dedicated root set selection algorithm. Section 6 provides simulation results demonstrating the proposed signal’s robustness against CFO. Section 7 concludes the paper.

## 2. Related Work

**Figure 1:**
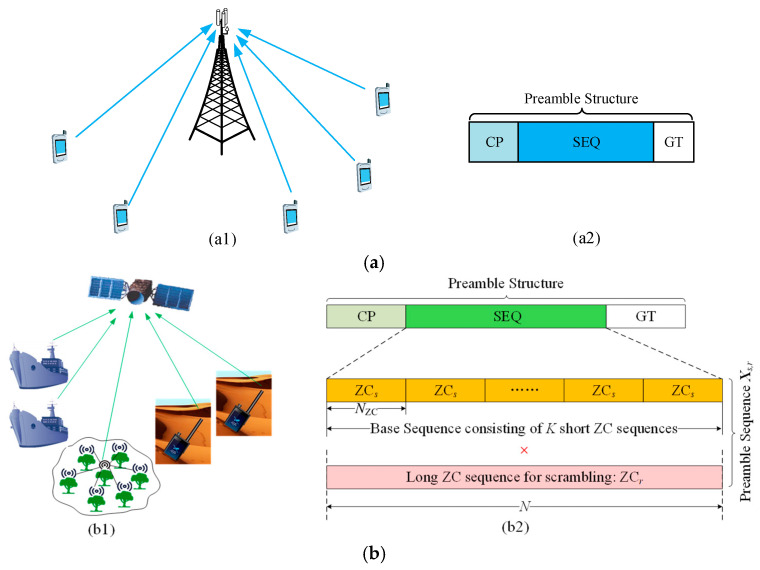
Illustration of random access signal structure. (**a**) Random access scenarios in TN (a1) and time-domain preamble signal structure in NR system (a2). (**b**) Random access scenarios in satellite communication systems (b1) and proposed preamble sequence structure adapted from [32] (b2).

Figure 1a illustrates the time-domain structure of the random access preamble signal in 4G LTE and 5G NR systems. Since the preamble transmission timing is derived from the downlink synchronization signals, the arrival time of the preamble at the base station includes a round-trip propagation delay (i.e., downlink plus uplink). Therefore, unlike the cyclic prefix (CP) in a normal orthogonal frequency division multiplexing (OFDM) symbol, which is primarily designed to address multipath delay spread, the CP of the random access preamble is extended to be sufficiently long to accommodate not only the channel delay spread but also the maximum round-trip propagation delay. This enables detection of the random access signal from a device without prior knowledge of its exact arrival timing. The guard time (GT) is introduced to prevent the random access signal from overlapping with the subsequent data symbol due to propagation delays. Since the following symbol contains uplink data transmissions from other devices, such overlap would cause interference and impair decoding performance.

A ZC sequence is used to generate the preamble sequence. It is defined as

(1) $${\mathrm{ZC}}_{\mu }=\left\{{\mathrm{ZC}}_{\mu }\left(n\right)=e^{-j\frac{\pi \mu n\left(n+1\right)}{N_{\mathrm{ZC}}}},n=0,1,\cdots ,N_{\mathrm{ZC}}-1\right\}$$

when the sequence length $N_{\mathrm{ZC}}$ is odd, or as

(2) $${\mathrm{ZC}}_{\mu }=\left\{{\mathrm{ZC}}_{\mu }\left(n\right)=e^{-j\frac{\pi \mu n^{2}}{N_{\mathrm{ZC}}}},n=0,1,\cdots ,N_{\mathrm{ZC}}-1\right\}$$

when $N_{\mathrm{ZC}}$ is even. The root $\mu \in \left\{1,2,\cdots ,N_{\mathrm{ZC}}-1\right\}$ must be coprime with the length $N_{\mathrm{ZC}}$. ZC sequences sharing the same root index but having different cyclic shifts are orthogonal, i.e.,

(3) $$\frac{1}{N_{\mathrm{ZC}}}\overset{N_{\mathrm{ZC}}-1}{\underset{n=0}{\sum }}{\mathrm{ZC}}_{\mu }\left(n+m_{1}\right){\mathrm{ZC}}_{\mu }^{*}\left(n+m_{2}\right)=\delta \left(m_{1}-m_{2}\right),m_{1},m_{2}=0,1,\cdots ,N_{\mathrm{ZC}}-1$$

Consequently, ZC sequences with the same root but distinct cyclic shifts are used to distinguish different random access preamble sequences. Sequences derived from distinct roots are introduced only when the number of available valid cyclic shifts for a single root is insufficient to support the required number of random access opportunities.

Figure 1b illustrates the structure of the proposed preamble in [32], which retains the CP-sequence-GT framework for compatibility with TNs while incorporating optimizations tailored for satellite communication systems. Specifically, the CP and GT durations are optimized to accommodate the maximum differential round-trip delay, assuming pre-compensation of CFO and common round-trip delay RTD across devices within the satellite beam coverage. In contrast to TN system designs, which rely on cyclic shifts to differentiate random access signals, this approach refrains from cyclic shift multiplexing. This decision arises because the maximum differential RTD in satellite systems exceeds the typical preamble duration of TN systems, rendering cyclic shifts ineffective for signal separation. To mitigate the significant path loss induced by long-haul satellite transmissions, the preamble sequence duration is extended to enhance signal energy. The specific duration is systematically determined by balancing key tradeoffs, including communication coverage requirements, TN compatibility constraints, and the maximum differential RTD [32].

The preamble sequence proposed in [32] concatenates K repetitions of a short ZC sequence, $\left\{{\mathrm{ZC}}_{s}\left(n\right),n=0,1,\cdots ,N_{\mathrm{ZC}}-1\right\}$, and scrambles the result with a long ZC sequence $\left\{{\mathrm{ZC}}_{r}\left(n\right),n=0,1,\cdots ,N-1\right\}$. This preamble sequence is mathematically defined as

(4) $$X_{s,r}=\left\{x_{s,r}\left(n\right)={\mathrm{ZC}}_{s}\left(n\right)\cdot {\mathrm{ZC}}_{r}\left(n\right),n=0,1,\cdots ,N-1\right\}$$

where $N=K\cdot N_{\mathrm{ZC}}$. Here, s and r denote the roots of the short and long ZC sequences, respectively. Distinct long ZC sequences with different roots r are assigned to different preamble sequences.

The deigned sequence exhibits ideal auto-correlation property, i.e.,

(5) $$\left|C_{r,r}\left(m\right)\right|=\left|\frac{1}{N}\overset{N-1}{\underset{n=0}{\sum }}x_{s,r}\left(n\right)\cdot x_{s,r}^{*}\left(n-m\right)\right|=\delta \left(m\right),m=0,1,\cdots ,N-1$$

provided $\gcd\left(r+sK,N\right)=1$. Here, the function $\gcd\left(r+sK,N\right)$ denotes the greatest common divisor of r+sK and N. The cross-correlation between distinct random access sequence $X_{s,r}$ and $X_{s,o}$ is

(6) $$\begin{array}{cc} \left|C_{r,o}\left(m\right)\right| & =\left|\frac{1}{N}\overset{N-1}{\underset{n=0}{\sum }}x_{s,r}\left(n\right)\cdot x_{s,o}^{*}\left(n-m\right)\right|\\ & =\left\{\begin{array}{c} \sqrt{\frac{g}{N}}\delta \left(d\right)\;N\;\mathrm{and}\;uv\;\mathrm{are}\;\mathrm{even},\mathrm{or}\;N\;\mathrm{is}\;\mathrm{odd},\\ \sqrt{\frac{g}{N}}\delta \left(d-\frac{g}{2}\right)\;N\;\mathrm{is}\;\mathrm{even}\;\mathrm{and}\;uv\;\mathrm{is}\;\mathrm{odd}, \end{array}\right. \end{array}$$

where $g≜\gcd\left(r-o,N\right)$, $u≜\frac{N}{g}$, $v≜\frac{r-o}{g}$.

To minimize the cross-correlation coefficient, it is necessary to reduce the value of g as much as possible. In [32], roots are carefully selected to satisfy g≤K and a pool of 128 random access signals are provided. Consequently, the upper bound on the cross-correlation magnitude is bounded by

(7) $$\left|C_{r,o}\left(m\right)\right|\leq \sqrt{\frac{g}{N}}\leq \sqrt{\frac{K}{KN_{\mathrm{ZC}}}}=\frac{1}{\sqrt{N_{\mathrm{ZC}}}}$$

## 3. Analyses of Frequency Offset Effect

The proposed design in [32] addresses most key requirements for satellite communication systems, including doubling the number of random access signals per PRACH resource compared to terrestrial LTE/NR systems, accounting for device-specific differential RTD, ensuring compatibility with terrestrial preamble architectures, and supporting broader coverage. However, it omits device-specific CFO considerations. Now, we will analyze the impact of CFO on this design, laying the foundation for developing CFO-resistant upgrades.

A device that wants to access the network randomly selects a preamble from the pool, for instance, a preamble P composed of a CP of length $N_{\mathrm{CP}}$ and a sequence $X_{s,r}$ of length N. This signal propagates through the channel with gain h and is received by the satellite receiver with a frequency offset Δf relative to the device. For mathematical tractability, the conventional block fading model is employed in the subsequent analysis, where the channel is assumed to remain constant throughout the transmission period.

At the satellite receiver, this preamble signal is sampled as

(8) $$\begin{array}{cc} y^{\prime }\left(n\right) & =hp\left(n-\tau \right)e^{j2\pi n\frac{\Delta f}{N_{\mathrm{ZC}}\Delta f_{\mathrm{sc}}}}+\omega \left(n\right)\\ & =hp\left(n-\tau \right)e^{j\frac{2\pi }{N}n\varepsilon }+\omega \left(n\right),n=0,1,\cdots ,N+N_{\mathrm{CP}}-1, \end{array}$$

where τ denotes the device-specific differential arrival delay, $\varepsilon ≜\frac{K\Delta f}{\Delta f_{\mathrm{sc}}}$ represents the normalized frequency offset, $\Delta f_{\mathrm{sc}}$ is the subcarrier spacing of the PRACH resource, and $\omega \left(n\right)∼CN\left(0,\sigma^{2}\right)$ is the complex-valued Gaussian noise with zero-mean and variance $\sigma^{2}$. For subsequent analyses, we define $\rho ≜\frac{{\left|h\right|}^{2}}{\sigma^{2}}$ as the received sample signal-to-noise ratio (SNR).

The receiver discards the first $N_{\mathrm{CP}}$ samples and retain only the last N samples from $y^{\prime }$. This results in y, which is defined as

(9) $$y\left(n\right)≜y^{\prime }\left(n+N_{\mathrm{CP}}\right)=hx_{s,r}\left(n-\tau \right)e^{j\frac{2\pi }{N}\varepsilon n}+w\left(n\right),\;n=0,\;1,\cdots ,\;N-1$$

where the constant phase $e^{j\frac{2\pi }{N}\varepsilon N_{\mathrm{CP}}}$ is incorporated into h for notational simplification, and $w\left(n\right)≜\omega \left(n+N_{\mathrm{CP}}\right)$.

The correlation between the received sequence and $X_{s,r}$ at hypothesis timing m is

(10) $$G(m,\varepsilon )=\frac{1}{N}\sum_{n=0}^{N-1}y\left(n\right)x_{s,r}^{*}\left(n-m\right)=hC_{r,r}(m,\varepsilon )+\varpi \left(m\right)$$

where $\varpi \left(m\right)=\frac{1}{N}\sum_{n=0}^{N-1}w\left(n\right)x_{s,r}^{*}\left(n-m\right)∼CN\left(0,\frac{\sigma^{2}}{N}\right)$,

(11) $$\begin{array}{cc} C_{r,r}\left(m,\varepsilon \right) & ≜\frac{1}{N}\sum_{n=0}^{N-1}\left[x_{s,r}\left(n-\tau \right)\cdot e^{j\frac{2\pi n\varepsilon }{N}}\right]x_{s,r}^{*}\left(n-m\right)\\ & =\frac{1}{N}e^{j\pi \frac{\left(r+sK\right)\left(m^{2}-\tau^{2}\right)-sK\left(m-\tau \right)+\left(\varepsilon -\left(r+Ks\right)\left(m-\tau \right)\right)\left(N-1\right)}{N}}\frac{\sin\left(\pi \left(\varepsilon -\left(r+Ks\right)\left(m-\tau \right)\right)\right)}{\sin\left(\frac{\pi \left(\varepsilon -\left(r+Ks\right)\left(m-\tau \right)\right)}{N}\right)}. \end{array}$$

In the presence of a frequency offset ε, the absolute value of this correlation is

(12) $$\left|C_{r,r}\left(m,\varepsilon \right)\right|=\frac{1}{N}\left|\frac{\sin\left(\pi \left(\varepsilon -\left(r+Ks\right)\left(m-\tau \right)\right)\right)}{\sin\left(\frac{\pi \left(\varepsilon -\left(r+Ks\right)\left(m-\tau \right)\right)}{N}\right)}\right|$$

Express $\varepsilon =\varepsilon_{i}+\varepsilon_{f}$, where $\varepsilon_{i}\in \mathbb{Z}$ and $\varepsilon_{f}\in (-0.5,0.5]$ denote the integer and the parts, respectively. For integer ε (i.e., $\varepsilon_{f}=0$ or $\varepsilon =\varepsilon_{i}$),

(13) $$\left|C_{r,r}\left(m,\varepsilon \right)\right|=\delta \left(m-\left(\varepsilon_{i}\cdot {\left(r+Ks\right)}^{-1}+\tau \right)\mathrm{mod}N\right)$$

This indicates that the integer $\varepsilon_{i}$ induces a cyclic shift in the peak correlation value. Specifically, it displaces the peak from $m^{†}=\tau$ to

(14) $$m^{†}=\left(\varepsilon_{i}\cdot {\left(r+Ks\right)}^{-1}+\tau \right)\mathrm{mod}N$$

This phenomenon obfuscates the precise disentanglement of τ and $\varepsilon_{i}$ from the observed peak position. Here, $a^{-1}$ denotes an integer within the range $\left[1,N-1\right]$ and satisfies $\left(a^{-1}\cdot a\right)\mathrm{mod}N=1$.

For a non-integer ε (i.e., $\varepsilon_{f}\neq 0$), it can be easily verified that $0<\left|C_{r,r}\left(m\neq m^{†},\varepsilon \right)\right|\leq \left|C_{r,r}\left(m=m^{†},\varepsilon \right)\right|<1$. This demonstrates that fractional ε not only attenuates the peak correlation amplitude at $m=m^{†}$ but also results in energy leakage across all the other cyclic shifts, complicating reliable peak detection and parameter estimation.

**Figure 2:**
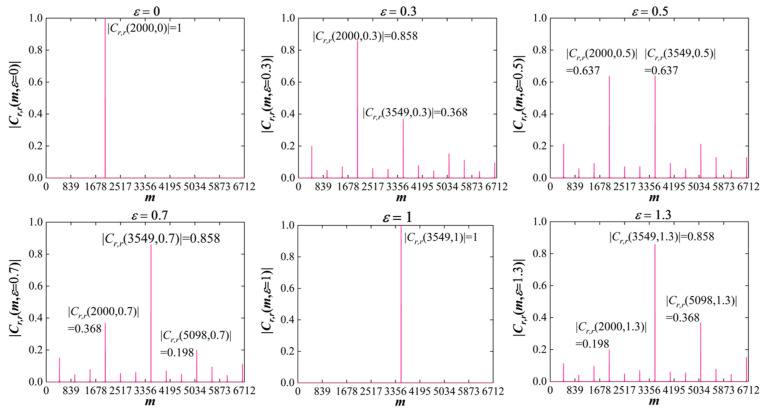
Illustration of $\left|C_{r,r}\left(m,\varepsilon \right)\right|$ under various frequency offsets, where s=1, r=5, K=8, $N_{\mathrm{ZC}}=839$, and τ=2000.

Figure 2 illustrates the correlation coefficient $\left|C_{r,r}\left(m,\varepsilon \right)\right|$ under various frequency offsets. The simulated parameters are s=1, r=5, K=8, $N_{\mathrm{ZC}}=839$, $N=K\cdot N_{\mathrm{ZC}}=6712$, and τ=2000. Here, $N_{\mathrm{ZC}}=839$ aligns with the length configuration used for terrestrial RACH in both 4G LTE and 5G NR, ensuring backward compatibility. Since ${\left(r+Ks\right)}^{-1}=1549$, the maximum correlation value is located at $m=m^{†}=\left(\varepsilon_{i}\cdot 1549+2000\right)\mathrm{mod}N$. For an integer ε, e.g., $\varepsilon =\varepsilon_{i}=1$, the correlation achieves the maximum value of one at $m=m^{†}=3549$, i.e., $\left|C_{r,r}\left(3549,1\right)\right|=1$, while it remains zero at all the other cyclic shifts, i.e., $\left|C_{r,r}\left(m\neq 3549,1\right)\right|=0$. For a non-integer ε, e.g., ε=0.7, where $\varepsilon_{i}=1$ and $\varepsilon_{f}=-0.3$, the maximum value decreases, e.g., $\left|C_{r,r}\left(3549,0.7\right)\right|=0.858<1$. Furthermore, the correlation value at other cyclic shifts becomes no longer zero, i.e., $\left|C_{r,r}\left(m\neq 3549,0.7\right)\right|>0$, which is referred to as energy leakage. Herein, energy leakage indicates the presence of non-zero correlation at cyclic shifts different from $m=m^{†}$.

As observed in Figure 2, for $-0.5<\varepsilon_{f}<0$, the majority of the leaked energy is located at

(15) $$m^{††}=\left(\left(\varepsilon_{i}-1\right)\cdot {\left(r+Ks\right)}^{-1}+\tau \right)\mathrm{mod}N=\left(m^{†}-{\left(r+Ks\right)}^{-1}\right)\mathrm{mod}N$$

Conversely, for $0<\varepsilon_{f}\leq 0.5$, the largest leaked energy shifts to

(16) $$m^{††}=\left(\left(\varepsilon_{i}+1\right)\cdot {\left(r+Ks\right)}^{-1}+\tau \right)\mathrm{mod}N=\left(m^{†}+{\left(r+Ks\right)}^{-1}\right)\mathrm{mod}N$$

In summary, when $0<\left|\varepsilon_{f}\right|<0.5$, the following inequality holds:

(17) $$0<\left|C_{r,r}\left(m\notin \left\{m^{††},m^{†}\right\},\varepsilon \right)\right|<\left|C_{r,r}\left(m^{††},\varepsilon \right)\right|<\left|C_{r,r}\left(m^{†},\varepsilon \right)\right|<1$$

A special case occurs when $\varepsilon_{f}=0.5$, at which the maximum correlation value is minimized. Simultaneously, the correlation value at $m^{††}$ equals that $m=m^{†}$, as described by

(18) $$0<\left|C_{r,r}\left(m\notin \left\{m^{†},m^{††}\right\},\varepsilon_{i}+0.5\right)\right|<\left|C_{r,r}\left(m^{††},\varepsilon_{i}+0.5\right)\right|=\left|C_{r,r}\left(m^{†},\varepsilon_{i}+0.5\right)\right|=0.637$$

For example, with ε=0.5 (i.e., $\varepsilon_{i}=0$), the maximum correlation occurs at both $m=m^{†}=2000$ and $m=m^{††}=3549$, i.e., $\left|C_{r,r}\left(2000,0.5\right)\right|=\left|C_{r,r}\left(3549,0.5\right)\right|=0.637$. At this point, the leaked energy attains its maximum value of approximately 0.637. In all other cases, the maximum leaked energy remains below 0.637.

**Figure 3:**
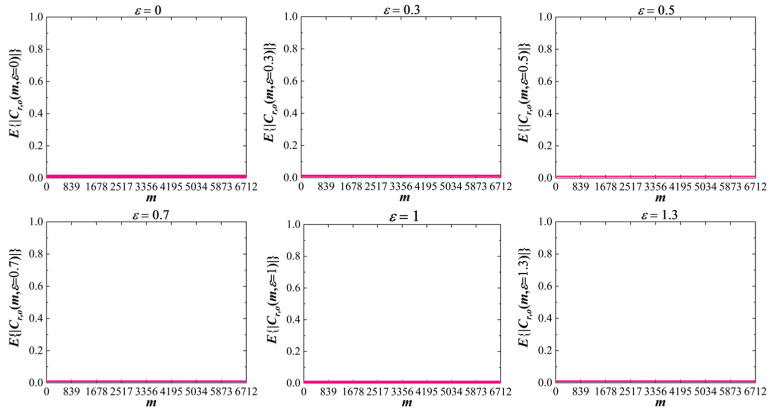
Illustration of $E\left\{\left|C_{r,o}\left(m,\varepsilon \right)\right|\right\}$ under various frequency offsets, where s=1, K=8, $N_{\mathrm{ZC}}=839$, τ=2000.

Figure 3 illustrates the average cross-correlation coefficient $E\left\{\left|C_{r,o}\left(m,\varepsilon \right)\right|\right\}$ of the proposed random access signals in [32] under varying CFOs. Here, $C_{r,o}\left(m,\varepsilon \right)$ denotes the cross-correlation between sequence $X_{s,r}$ and $X_{s,o}$ with CFO ε, i.e.,

(19) $$C_{r,o}\left(m,\varepsilon \right)=\frac{1}{N}\sum_{n=0}^{N-1}x_{s,r}\left(n-\tau \right)\cdot e^{j\frac{2\pi n\varepsilon }{N}}x_{s,o}^{*}\left(n-m\right)$$

The parameters for Figure 3 employs s=1, r=5, K=8, $N_{\mathrm{ZC}}=839$, and $N=K\cdot N_{\mathrm{ZC}}=6712$. Roots r and o (designed to be distinct) are from Table 4 in [32] to differentiate random access sequences. Results demonstrate that the proposed sequences maintain low cross-correlation performance, exhibiting robustness against CFO effects.

## 4. Upgraded CFO-Resistant Random Access Sequence Design and Detection

Section 3 analyzed the frequency offset effect on the preamble designed in [32] for satellite systems. Two primary conclusions are drawn: First, the integer CFO component $\varepsilon_{i}$ shifts the correlation peak position to $m=m^{†}$. Second, the fractional CFO component $\varepsilon_{f}$ attenuates the peak magnitude at $m=m^{†}$ below 1 and causes energy leakage to other cyclic shifts, with the dominant leakage occurring at $m=m^{††}$. To ensure reliable detection performance, including timing estimation for uplink transmission, a CFO-resistant preamble design and its detection procedure are proposed.

### 4.1. Upgraded Random Access Sequence Design

The preamble design in [32] is now modified to adapt to device-specific differential frequency offsets. Since both τ and $\varepsilon_{i}$ in (14) are unknown, deriving these parameters requires two independent linear equations. A straightforward solution is to generate a second equation analogous to (14).

Consequently, a pair of sequences from [32], denoted as $X_{s,r_{1}}$ and $X_{s,r_{2}}$, are combined to form a new random access sequence X:

(20) $$x\left(n\right)=\frac{1}{\sqrt{2}}\left(x_{s,r_{1}}\left(n\right)+x_{s,r_{2}}\left(n\right)\right)=\frac{1}{\sqrt{2}}\left({\mathrm{ZC}}_{s}\left(n\right)\cdot {\mathrm{ZC}}_{r_{1}}\left(n\right){+\mathrm{ZC}}_{s}\left(n\right)\cdot {\mathrm{ZC}}_{r_{2}}\left(n\right)\right)$$

The received sequence is then expressed as

(21) $$\begin{array}{ll} y(n) & =hx\left(n-\tau \right)\cdot e^{j\frac{2\pi n\varepsilon }{N}}+w(n)\\ & =\frac{h}{\sqrt{2}}\left(x_{s,r_{1}}\left(n-\tau \right)+x_{s,r_{2}}\left(n-\tau \right)\right)e^{j\frac{2\pi }{N}\varepsilon n}+w\left(n\right)\;,\;n=0,\;1,\cdots ,\;N\;-1. \end{array}$$

At the receiver, correlations are performed between $\left\{y(n)\right\}$ and each reference sequence $X_{s,r_{1}}$ and $X_{s,r_{2}}$, i.e.,

(22) $$G_{1}(m,\varepsilon )=\frac{1}{N}\sum_{n=0}^{N-1}y\left(n-\tau \right)x_{s,r_{1}}^{*}\left(n-m\right)=\frac{h}{\sqrt{2}}\left[C_{r_{1},r_{1}}\left(m,\varepsilon \right)+C_{r_{2},r_{1}}\left(m,\varepsilon \right)\right]+\varpi_{r_{1}}\left(m\right)$$

and

(23) $$G_{2}(m,\varepsilon )=\frac{1}{N}\sum_{n=0}^{N-1}y\left(n-\tau \right)x_{s,r_{2}}^{*}\left(n-m\right)=\frac{h}{\sqrt{2}}\left[C_{r_{2},r_{2}}\left(m,\varepsilon \right)+C_{r_{1},r_{2}}\left(m,\varepsilon \right)\right]+\varpi_{r_{2}}\left(m\right)$$

where $C_{r_{2},r_{1}}\left(m,\varepsilon \right)=\frac{1}{N}\sum_{n=0}^{N-1}x_{s,r_{2}}\left(n-\tau \right)\cdot e^{j\frac{2\pi n\varepsilon }{N}}x_{s,r_{1}}^{*}\left(n-m\right)$, $\varpi_{r_{1}}\left(m\right)∼CN\left(0,\frac{\sigma^{2}}{N}\right)$, $C_{r_{1},r_{2}}\left(m,\varepsilon \right)=\frac{1}{N}\sum_{n=0}^{N-1}x_{s,r_{1}}\left(n-\tau \right)\cdot e^{j\frac{2\pi n\varepsilon }{N}}x_{s,r_{2}}^{*}\left(n-m\right)$, $\varpi_{r_{2}}\left(m\right)∼CN\left(0,\frac{\sigma^{2}}{N}\right)$.

### 4.2. Upgraded Random Access Sequence Detection Mechanism

The complex outputs $G_{1}(m,\varepsilon )$ and $G_{2}(m,\varepsilon )$ are converted to magnitude-squared metrics, defined as $\eta_{1}\left(m,\varepsilon \right)≜{\left|G_{1}(m,\varepsilon )\right|}^{2}$ and $\eta_{2}\left(m,\varepsilon \right)≜{\left|G_{2}(m,\varepsilon )\right|}^{2}$. This conversion removes phase information, retaining only magnitude for detection.

The baseline detection metric is defined as

(24) $$\lambda \left(\varepsilon \right)=\eta_{1}\left(q_{1},\varepsilon \right)+\eta_{2}\left(q_{2},\varepsilon \right)$$

where

(25) $$q_{1}=\underset{m\in \left[0,N-1\right]}{\arg\max}\eta_{1}\left(m,\varepsilon \right),q_{2}=\underset{m\in \left[0,N-1\right]}{\arg\max}\eta_{2}\left(m,\varepsilon \right)$$

In the presence of an integer ε, it follows that $q_{1}=m_{1}^{†}=\left(\varepsilon_{i}\cdot {\left(r_{1}+Ks\right)}^{-1}+\tau \right)\mathrm{mod}N$ and $q_{2}=m_{2}^{†}=\left(\varepsilon_{i}\cdot {\left(r_{2}+Ks\right)}^{-1}+\tau \right)\mathrm{mod}N$ under noiseless conditions. As demonstrated in Figure 2 and Figure 3, cross-correlation values are significantly lower than the peak auto-correlation and are negligible compared to the dominate peak auto-correlation. Optimal detection is achieved in this case because $\left|C_{r,r}\left(m^{†},\varepsilon_{i}\right)\right|=1$ and $\left|C_{r,r}\left(m\neq m^{†},\varepsilon_{i}\right)\right|=0$. Consequently, an integer CFO introduces no detection loss but results in a timing offset.

For non-integer CFO ($\varepsilon_{f}\neq 0$), peak attenuation ($\left|C_{r,r}\left(m=m^{†},\varepsilon \neq \varepsilon_{i}\right)\right|<1$) and energy leakage ($\left|C_{r,r}\left(m\neq m^{†},\varepsilon \neq \varepsilon_{i}\right)\right|>0$) degrades detection performance. To mitigate this, the dominate leakage energy at $m=m^{††}$ can be incorporated into a revised detection metric:

(26) $$\lambda \left(\varepsilon \right)=\eta_{1}\left(q_{1},\varepsilon \right)+\eta_{1}\left(v_{1},\varepsilon \right)+\eta_{2}\left(q_{2},\varepsilon \right)+\eta_{2}\left(v_{2},\varepsilon \right)$$

where leakage positions $v_{1}$ and $v_{2}$ correspond to secondary maxima excluding primary peaks, formally defined as

(27) $$v_{1}=\underset{\begin{array}{l} m\in \left[0,N-1\right]\\ m\neq q_{1} \end{array}}{\arg\max}\eta_{1}\left(m,\varepsilon \right),v_{2}=\underset{\begin{array}{l} m\in \left[0,N-1\right]\\ m\neq q_{2} \end{array}}{\arg\max}\eta_{2}\left(m,\varepsilon \right)$$

Ignoring noise, the positions $\left\{v_{1},v_{2}\right\}$ are

(28) $$v_{1}=m_{1}^{††}=\left(m_{1}^{†}-{\left(r_{1}+Ks\right)}^{-1}\right)\mathrm{mod}N,v_{2}=m_{2}^{††}=\left(m_{2}^{†}-{\left(r_{2}+Ks\right)}^{-1}\right)\mathrm{mod}N$$

for $-0.5<\varepsilon_{f}<0$. Otherwise, i.e., $0<\varepsilon_{f}\leq 0.5$,

(29) $$v_{1}=m_{1}^{††}=\left(m_{1}^{†}+{\left(r_{1}+Ks\right)}^{-1}\right)\mathrm{mod}N,v_{2}=m_{2}^{††}=\left(m_{2}^{†}+{\left(r_{2}+Ks\right)}^{-1}\right)\mathrm{mod}N$$

Sequence X is declared detected if $\lambda \left(\varepsilon \right)>T$, where the threshold T is set to maintain a reasonably false alarm rate, e.g., 0.1% [32].

### 4.3. Timing Estimation in the Presence of CFO

The preamble indicates device presence and enables uplink timing estimation (differential round-trip propagation delay) at the satellite receiver. This section details timing estimation post-detection.

#### 4.3.1. Integer CFO Case ($\varepsilon_{f}=0$)

When $\varepsilon_{f}=0$ (ignoring noise), $\left|C_{r,r}\left(m\neq m^{†},\varepsilon =\varepsilon_{i}\right)\right|=0$. Thus, $m^{††}$ does not exist due to the absence of energy leakage. In this case, the time offset τ must therefore be estimated solely from the peak correlation positions $m_{1}^{†}$ and $m_{2}^{†}$. However, the modulo operation in $m^{†}$ as shown in (14) prevents the direct extraction of τ and ε. Define $\Delta m^{†}\left(\varepsilon_{i}\right)$ as

(30) $$\Delta m^{†}\left(\varepsilon_{i}\right)≜\left(m_{1}^{†}-m_{2}^{†}\right)\mathrm{mod}N=\left(\varepsilon_{i}\cdot \Delta_{r_{1},r_{2}}\right)\mathrm{mod}N$$

where $\Delta_{r_{1},r_{2}}≜{\left(r_{1}+Ks\right)}^{-1}-{\left(r_{2}+Ks\right)}^{-1}$ is a constant. It is apparent that $\Delta m^{†}\left(\varepsilon_{i}\right)$ depends solely on $\varepsilon_{i}$, not τ. If a one-to-one mapping exists between $\varepsilon_{i}$ and $\Delta m^{†}\left(\varepsilon_{i}\right)$, then τ can be obtained from

(31) $$\tau =\left(m^{†}-\varepsilon_{i}\cdot {\left(r+Ks\right)}^{-1}\right)\mathrm{mod}N$$

provided $\varepsilon_{i}$ is uniquely determined by $\Delta m^{†}\left(\varepsilon_{i}\right)$.

This mapping requires all elements in set $Q_{r_{1},r_{2}}$ to be unique. The set $Q_{r_{1},r_{2}}$ is defined as

(32) $$Q_{r_{1},r_{2}}≜\left\{Q_{r_{1},r_{2}}\left(n\right),0\leq n\leq L-1\right\}=\left\{\left(\left(n-\zeta \right)\cdot \Delta_{r_{1},r_{2}}\right)\mathrm{mod}N,0\leq n\leq L-1\right\}$$

where ζ is the maximum integer CFO ($-\zeta \leq \varepsilon_{i}\leq \zeta$), L=2ζ+1 is the number of elements in set $Q_{r_{1},r_{2}}$. Element uniqueness is equivalent to the requirement that

(33) $$\left(\Delta n\cdot \Delta_{r_{1},r_{2}}\right)\mathrm{mod}N\neq 0$$

for $1\leq \left|\Delta n\right|\leq L-1$.

In this case, timing estimation can be performed in three steps. First, obtain $q_{1}$ and $q_{2}$ from (25) and compute $\Delta q≜\left(q_{1}-q_{2}\right)\mathrm{mod}N$. Second, find index n of Δq in set $Q_{r_{1},r_{2}}$ such that $Q_{r_{1},r_{2}}\left(n\right)=\Delta q$. Then, the integer CFO can be determined as $\varepsilon_{i}=n-\zeta$. Third, estimate τ via (31).

#### 4.3.2. Non-Integer CFO Case ($\varepsilon_{f}\neq 0$)

For non-integer CFO where $0<\left|\varepsilon_{f}\right|<0.5$, the aforementioned method remains effective because the cyclic shift $m=m^{†}$ consistently retains the peak correlation in this case. However, when $\varepsilon_{f}=0.5$, the correlation strengths at positions $m^{†}$ and $m^{††}$ become equal, $\left|C_{r,r}\left(m^{††},\varepsilon =\varepsilon_{i}+0.5\right)\right|=\left|C_{r,r}\left(m^{†},\varepsilon =\varepsilon_{i}+0.5\right)\right|$, rendering the two positions indistinguishable. Consequently, q in (25) (and v in (29)) could correspond to either $m^{†}$ and $m^{††}$, invalidating the prior timing method. A robust method is therefore required for $\varepsilon_{f}\neq 0$, including $\varepsilon_{f}=0.5$.

Consider the characteristics of $m_{1}^{††}$ and $m_{2}^{††}$. It is observed that

(34) $$\begin{array}{cc} \left(m_{1}^{††}-m_{2}^{††}\right)\mathrm{mod}N & =\left\{\begin{array}{c} \left(\left(\varepsilon_{i}-1\right)\cdot \Delta_{r_{1},r_{2}}\right)\mathrm{mod}N,-0.5<\varepsilon_{f}<0\\ \left(\left(\varepsilon_{i}+1\right)\cdot \Delta_{r_{1},r_{2}}\right)\mathrm{mod}N,0<\varepsilon_{f}\leq 0.5 \end{array}\right.\\ & =\left\{\begin{array}{c} \Delta m^{†}\left(\varepsilon_{i}-1\right),-0.5<\varepsilon_{f}<0,\\ \Delta m^{†}\left(\varepsilon_{i}+1\right),0<\varepsilon_{f}\leq 0.5. \end{array}\right. \end{array}$$

Assuming (33) holds, the differential leakage positions also exhibit distinct mappings for different $\varepsilon_{i}$. It can be seen that $\left(m_{1}^{††}-m_{2}^{††}\right)\mathrm{mod}N$ corresponds to $\Delta m^{†}\left(\varepsilon_{i}-1\right)$ for $-0.5<\varepsilon_{f}<0$ and to $\Delta m^{†}\left(\varepsilon_{i}+1\right)$ for $0<\varepsilon_{f}\leq 0.5$. Timing estimation can proceed analogously using these mapped values, i.e.,

(35) $$\hat{\tau }=\left\{\begin{array}{c} \left(m^{††}-\left(\varepsilon_{i}-1\right)\cdot {\left(r+Ks\right)}^{-1}\right)\mathrm{mod}N,-0.5<\varepsilon_{f}<0,\\ \left(m^{††}-\left(\varepsilon_{i}+1\right)\cdot {\left(r+Ks\right)}^{-1}\right)\mathrm{mod}N,0<\varepsilon_{f}\leq 0.5. \end{array}\right.$$

Define the set $V_{r_{1},r_{2}}$ to include all the possible $\left(m_{1}^{††}-m_{2}^{††}\right)\mathrm{mod}N$. Thus,

(36) $$\begin{array}{cc} V_{r_{1},r_{2}} & ≜\left\{\left(m_{1}^{††}-m_{2}^{††}\right)\mathrm{mod}N\right\}\\ & =\left\{\begin{array}{c} V_{r_{1},r_{2}}^{1},-0.5<\varepsilon_{f}<0\\ V_{r_{1},r_{2}}^{2},0<\varepsilon_{f}\leq 0.5 \end{array}\right.\\ & =\left\{\begin{array}{c} \left\{\Delta m^{†}\left(\varepsilon_{i}-1\right),-\zeta \leq \varepsilon_{i}\leq \zeta \right\},-0.5<\varepsilon_{f}<0\\ \left\{\Delta m^{†}\left(\varepsilon_{i}+1\right)\mathrm{mod}N,-\zeta \leq \varepsilon_{i}\leq \zeta \right\},0<\varepsilon_{f}\leq 0.5 \end{array}\right.\\ & =\left\{\begin{array}{c} \left\{Q_{r_{1},r_{2}}\left(n-1\right),0\leq n\leq L-1\right\},-0.5<\varepsilon_{f}<0,\\ \left\{Q_{r_{1},r_{2}}\left(n+1\right),0\leq n\leq L-1\right\},0<\varepsilon_{f}\leq 0.5. \end{array}\right. \end{array}$$

Define the composite set $QV_{r_{1},r_{2}}$ as the union of the fundamental position difference set $Q_{r_{1},r_{2}}$ and leakage-induced position difference sets $V_{r_{1},r_{2}}^{1}$ and $V_{r_{1},r_{2}}^{2}$, i.e.,

(37) $$QV_{r_{1},r_{2}}≜Q_{r_{1},r_{2}}\cup V_{r_{1},r_{2}}^{1}\cup V_{r_{1},r_{2}}^{2}=\left\{\left(\left(n-\zeta -1\right)\cdot \Delta_{r_{1},r_{2}}\right)\mathrm{mod}N,0\leq n\leq L+1\right\}$$

This set contains all possible values from both the peak position differences $\left\{\left(m_{1}^{†}-m_{2}^{†}\right)\mathrm{mod}N\right\}$ and the leakage position differences $\left\{\left(m_{1}^{††}-m_{2}^{††}\right)\mathrm{mod}N\right\}$.

Given that the CFO ε is inherently unknown at the satellite receiver, a uniform timing estimation procedure must operate independently of whether the primary peak positions $\left\{m_{1}^{†},m_{2}^{†}\right\}$ or secondary leakage components $\left\{m_{1}^{††},m_{2}^{††}\right\}$ are utilized, provided the uniqueness condition for elements in $QV_{r_{1},r_{2}}$ holds. This leads to:

**Proposition 1.**  *To enable uniform timing estimation using either the primary peak positions* $\left\{m_{1}^{†},m_{2}^{†}\right\}$ *or secondary leakage positions* $\left\{m_{1}^{††},m_{2}^{††}\right\}$*, the roots* $\left\{r_{1},r_{2}\right\}$ *of the proposed random access sequence* X *in (20) must satisfy* $QV_{r_{1},r_{2}}\left(n_{1}\right)\neq QV_{r_{1},r_{2}}\left(n_{2}\right)$ *for all distinct index pairs* $n_{1}$*,* $n_{2}\in \left[0,L+1\right]$ *(*$n_{1}\neq n_{2}$*).*

Meanwhile, the cross-differences $\left(m_{1}^{†}-m_{2}^{††}\right)\mathrm{mod}N$ and $\left(m_{1}^{††}-m_{2}^{†}\right)\mathrm{mod}N$ must not interfere with timing estimation. Define sets $A_{r_{1},r_{2}}$ and $B_{r_{1},r_{2}}$ as

(38) $$\begin{array}{cc} A_{r_{1},r_{2}} & ≜\left\{\left(m_{1}^{†}-m_{2}^{††}\right)\mathrm{mod}N\right\}\\ & =\left\{\begin{array}{c} \left\{\left(\varepsilon_{i}\cdot \Delta_{r_{1},r_{2}}+{\left(r_{2}+Ks\right)}^{-1}\right)\mathrm{mod}N,-\zeta \leq \varepsilon_{i}\leq \zeta \right\},-0.5<\varepsilon_{f}<0\\ \left\{\left(\varepsilon_{i}\cdot \Delta_{r_{1},r_{2}}-{\left(r_{2}+Ks\right)}^{-1}\right)\mathrm{mod}N,-\zeta \leq \varepsilon_{i}\leq \zeta \right\},0<\varepsilon_{f}\leq 0.5 \end{array}\right.\\ & ≜\left\{\begin{array}{c} A_{r_{1},r_{2}}^{1},-0.5<\varepsilon_{f}<0,\\ A_{r_{1},r_{2}}^{2},0<\varepsilon_{f}\leq 0.5, \end{array}\right. \end{array}$$

(39) $$\begin{array}{cc} B_{r_{1},r_{2}} & ≜\left\{\left(m_{1}^{††}-m_{2}^{†}\right)\mathrm{mod}N\right\}\\ & =\left\{\begin{array}{c} \left\{\left(\varepsilon_{i}\cdot \Delta_{r_{1},r_{2}}-{\left(r_{1}+Ks\right)}^{-1}\right)\mathrm{mod}N,-\zeta \leq \varepsilon_{i}\leq \zeta \right\},-0.5<\varepsilon_{f}<0\\ \left\{\left(\varepsilon_{i}\cdot \Delta_{r_{1},r_{2}}+{\left(r_{1}+Ks\right)}^{-1}\right)\mathrm{mod}N,-\zeta \leq \varepsilon_{i}\leq \zeta \right\},0<\varepsilon_{f}\leq 0.5 \end{array}\right.\\ & ≜\left\{\begin{array}{c} B_{r_{1},r_{2}}^{1},-0.5<\varepsilon_{f}<0,\\ B_{r_{1},r_{2}}^{2},0<\varepsilon_{f}\leq 0.5. \end{array}\right. \end{array}$$

Thus, we have:

**Proposition 2.**  *To avoid interference from these cross-differences, it requires that* $QV_{r_{1},r_{2}}\cap AB_{r_{1},r_{2}}=\emptyset$*, where* $AB_{r_{1},r_{2}}=A_{r_{1},r_{2}}^{1}\cup A_{r_{1},r_{2}}^{2}\cup B_{r_{1},r_{2}}^{1}\cup B_{r_{1},r_{2}}^{2}$.

Consequently, a uniform timing estimation procedure can be performed, as summarized subsequently.

#### 4.3.3. Robust Timing Estimation Procedure

At the satellite receiver, the detection of a potential random access sequence X initiates with cyclic shift correlation operation between the received sequence and the paired reference sequences $X_{s,r_{1}}$ and $X_{s,r_{2}}$. This processing yields peak positions $q_{1}$, $q_{2}$ (c.f. (25)) and leakage positions $v_{1}$, $v_{2}$ (c.f. (27)). Sequence X is declared detected when the composite detection metric satisfies $\lambda \left(\varepsilon \right)>T$.

Following successful detection, timing estimation proceeds using the identified positions $q_{1}$, $v_{1}$, $q_{2}$, and $v_{2}$. Position sets $E_{r_{1},r_{2}}$ and $F_{r_{1},r_{2}}$ are defined as

(40) $$E_{r_{1},r_{2}}=\left\{q_{1},v_{1},q_{1},v_{1}\right\},F_{r_{1},r_{2}}=\left\{q_{2},v_{2},v_{2},q_{2}\right\}$$

where element duplication facilitates subsequent element-wise operations. The difference set is computed as

(41) $$\begin{array}{cc} Z_{r_{1},r_{2}} & =\left(E_{r_{1},r_{2}}-F_{r_{1},r_{2}}\right)\mathrm{mod}N\\ & =\left\{\left(q_{1}-q_{2}\right)\mathrm{mod}N,\left(v_{1}-v_{2}\right)\mathrm{mod}N,\left(q_{1}-v_{2}\right)\mathrm{mod}N\right\},\left(v_{1}-q_{2}\right)\mathrm{mod}N. \end{array}$$

Under ideal noiseless conditions and ignoring permutation order, the elements of set $Z_{r_{1},r_{2}}$ correspond to $\left(m_{1}^{†}-m_{2}^{†}\right)\mathrm{mod}N$, $\left(m_{1}^{††}-m_{2}^{††}\right)\mathrm{mod}N$, $\left(m_{1}^{†}-m_{2}^{††}\right)\mathrm{mod}N$, and $\left(m_{1}^{††}-m_{2}^{†}\right)\mathrm{mod}N$. This means at least one element $Z_{r_{1},r_{2}}\left(i\right)$ satisfies $Z_{r_{1},r_{2}}\left(i\right)\in QV_{r_{1},r_{2}}$ for $i\in \left[0,3\right]$ irrespective of integer or non-integer CFO. It enables implementation of a unified timing estimation procedure without CFO knowledge prerequisites.

The receiver performs index mapping by comparing $Z_{r_{1},r_{2}}\left(i\right)$ to the predefined set $QV_{r_{1},r_{2}}$, identifying the specific index n (0≤n≤L+1) such that $QV_{r_{1},r_{2}}\left(n\right)=Z_{r_{1},r_{2}}\left(i\right)$. The device-specific differential delay is subsequently estimated as

(42) $$\hat{\tau }=\left(E_{r_{1},r_{2}}\left(i\right)-\left(n-\zeta -1\right)\cdot {\left(r_{1}+Ks\right)}^{-1}\right)\mathrm{mod}N$$

Note that noise may cause $Z_{r_{1},r_{2}}\cap QV_{r_{1},r_{2}}=\emptyset$, indicating timing estimation failure.

The complete detection and estimation framework is illustrated in Algorithm 1. Successful random access requires simultaneous satisfaction of two conditions: (1) detection threshold exceedance ($\lambda \left(\varepsilon \right)>T$), and (2) accurate delay estimation ($\hat{\tau }=\tau$).
**Algorithm 1** The detection and timing estimation algorithm for random access sequence X
**Input**: received signal y, random access sequence X, threshold T, maximum integer CFO ζ**Output**: detection indicator d, estimated timing $\hat{\tau }$1: Calculate $q_{1}$ and $q_{2}$ using (25), $v_{1}$ and $v_{2}$ using (27), and $\lambda \left(\varepsilon \right)$ using (26).2: **if** $\lambda \left(\varepsilon \right)<T$ **then** d=0, $\hat{\tau }=\emptyset$3: **else**
4:       calculate $QV_{r_{1},r_{2}}$ using (37), $Z_{r_{1},r_{2}}$ using (41)5:         **if** $Z_{r_{1},r_{2}}\cap QV_{r_{1},r_{2}}=\emptyset$ **then** d=0, $\hat{\tau }=\emptyset$6:         **else**7:                *d* = 18:                **for** *i* = 0 to 3 **do**9:                      **if** $Z_{r_{1},r_{2}}\left(i\right)\in QV_{r_{1},r_{2}}$ **then**10:                          identify index n (0≤n≤L+1) where $QV_{r_{1},r_{2}}\left(n\right)=Z_{r_{1},r_{2}}\left(i\right)$,11:                          and calculate $\hat{\tau }$ using (42)12:                          break13:                    **end if**14:              **end for**15:       **end if**16: **end if**

## 5. Root Selection Algorithm

Root sequence selection criteria for optimal performance are derived in this section. The requirements for selecting roots s, $r_{1}$, and $r_{2}$ to construct a random access sequence $X=\frac{1}{\sqrt{2}}\left(X_{s,r_{1}}+X_{s,r_{2}}\right)$ are established, where $r_{1}=R_{\mathrm{root}1}\left(i\right)$ and $r_{2}=R_{\mathrm{root}2}\left(i\right)$, with $R_{\mathrm{root}}=R_{\mathrm{root}1}\cup R_{\mathrm{root}2}$ defining the unified root set. Here, $i\in \left[1,I\right]$ is the index of the random access sequence in the pool, I denotes the maximum number of random access preamble sequences required per beam coverage. Six critical conditions govern the root selection process. First, the root s of the short ZC sequence, with $s\in \left[1,N_{\mathrm{ZC}}-1\right]$, must be relatively prime to its length $N_{ZC}$. Second, the root r of the long ZC sequence, with $r\in \left[1,N-1\right]$ and $r\in R_{\mathrm{root}}$, must be relatively prime to its sequence length N. Both Requirements 1 and 2 are mandated by the definition of the ZC sequence. Third, the expression r+sK must be relatively prime to N for all $r\in R_{\mathrm{root}}$ to optimize the auto-correlation of the sequence $X_{s,r}$. Fourth, for any distinct pair $r,o\in R_{\mathrm{root}}$ with r>o, the greatest common divisor $g=\mathrm{GCD}\left(r-o,N\right)$ must satisfy g≤K, as referenced in Equation (7), to minimize the cross-correlation. Fifth, for a pair of roots $r_{1}$ and $r_{2}$ intended for sequence construction, $QV_{r_{1},r_{2}}\left(n\right)$ must be distinct for all $n_{1}$, $n_{2}\in \left[0,L+1\right]$ with $n_{1}\neq n_{2}$, i.e., $QV_{r_{1},r_{2}}\left(n_{1}\right)\neq QV_{r_{1},r_{2}}\left(n_{2}\right)$, as derived in Proposition 1. Finally, the pair $\left\{r_{1},r_{2}\right\}$ must satisfy $QV_{r_{1},r_{2}}\cap AB_{r_{1},r_{2}}=\emptyset$, as specified in Proposition 2. The final two requirements are used to guarantee the timing estimation performance in the presence of CFO.

The systematic selection algorithm for $R_{\mathrm{root}1}$ and $R_{\mathrm{root}2}$ is illustrated in Algorithm 2. To simplify implementation, the short ZC root s can be fixed at s=1, inherently fulfilling Requirement 1. The algorithm iteratively evaluates all roots $\left\{r\left|r\in \left[1,N-1\right]\right.\right\}$ in ascending order, admitting candidates to $R_{\mathrm{root}1}$ or $R_{\mathrm{root}2}$ only upon satisfying Requirements 2–6. An intermediate variable $g^{\prime }\in \left[1,K\right]$ is employed to constrain the upper bound of g, thereby minimizing the cross-correlation.
**Algorithm 2** Root selection algorithm in the presence of CFO**Initialization**: *N*, *K*, *I*, *s* = 1, $R_{\mathrm{root}1}\left[1:I\right]=\emptyset$, $R_{\mathrm{root}2}\left[1:I\right]=0$, $R_{\mathrm{root}}\left[1:2I\right]=\emptyset$, $g^{\prime }=1$, *r* = 1, *i* = 1, *j* = 11: **While** i≤2I **do**2:       **if** r≥N **then** $g^{\prime }=g^{\prime }+1$, *r* = 1, *i* = 1, *j* = 1, $R_{\mathrm{root}1}\left[1:I\right]=\emptyset$, $R_{\mathrm{root}2}\left[1:I\right]=0$, $R_{\mathrm{root}}\left[1:2I\right]=\emptyset$3:       **else**4:            **if**
$\gcd\left(r,N\right)\neq 1$ or $\gcd\left(r+sK,N\right)\neq 1$ **then**
*r* = *r* + 15:            **else**
6:                    **if** *i*=1 **then**
$R_{\mathrm{root}1}\left[j\right]=r$, *j* = *j* + 1, $R_{\mathrm{root}}\left[i\right]=r$, *i* = *i* + 1, *r* = *r* + 17:                     **else**
8:                            **if** there exists $\mu \in R_{\mathrm{root}}$ such that $\gcd\left(N,r-\mu \right)>g^{\prime }$ **then**
*r* = *r* + 19:                            **else**10:                                   $r_{2}=r$, *index* = 011:                                   **for** *k* = 1 to *j*-1 **do**
12:                                         **if** $R_{\mathrm{root}2}\left[k\right]=0$ **then**13:                                         $r_{1}=R_{\mathrm{root}1}\left[k\right]$14:                                                 **if**
$QV_{r_{1},r_{2}}\left(n_{1}\right)\neq QV_{r_{1},r_{2}}\left(n_{2}\right)$ for all $n_{1}\neq n_{2}$ ($n_{1}$, $n_{2}\in \left[0,L+1\right]$), 15:                                                 and $QV_{r_{1},r_{2}}\cap AB_{r_{1},r_{2}}=\emptyset$ **then**
16:                                                         $R_{\mathrm{root}2}\left[k\right]=r$, $R_{\mathrm{root}}\left[i\right]=r$, *i* = *i* + 1, *r* = *r* + 1, *index* = 117:                                                         **break**18:                                                 **end if**19:                                         **end if**20:                                   **end for**21:                                   **if** *index* = 0 and j≤I **then**
22:                                   $R_{\mathrm{root}1}\left[j\right]=r$, *j* = *j* + 1, $R_{\mathrm{root}}\left[i\right]=r$, *i* = *i* + 1, *r* = *r* + 123:                                   **end if**24:                          **end if**25:                  **end if**26:          **end if**27:     **end if**28: **end while**

## 6. Simulation Results

This section presents the detection performance evaluated through numerical simulations. The detection of random access sequences is conducted using Algorithm 1, with the common Doppler shift and round-trip delay (RTD) assumed to be calculated and eliminated by the satellite. Consequently, only the device-specific RTD and CFO require consideration. In the simulated scenario, an access device moves at 350 km/h at a carrier frequency of 2 GHz, resulting in a device-specific Doppler frequency of 648 Hz. Since the frequency synthesizer is synchronized to the downlink synchronization channel, the Doppler effect is thus doubled at the uplink receiver, resulting in a frequency offset of 1296 Hz. The frequency synthesizer error, typically within 0.1 ppm or 200 Hz at 2 GHz [32], contributes to a total frequency offset of 1496 Hz at the receiver. Consequently, the normalized CFO ε falls within the range $\left[-\frac{K\left|\Delta f\right|}{\Delta f_{\mathrm{sc}}},\frac{K\left|\Delta f\right|}{\Delta f_{\mathrm{sc}}}\right]=\left[-9.57,9.57\right]$, where the subcarrier spacing $\Delta f_{\mathrm{sc}}=1250$ Hz and K=8 [32]. The maximum integer frequency offset ζ is 10, i.e., $-10\leq \varepsilon_{i}\leq 10$.

The root pairs $\left\{r_{1},r_{2}\right\}$ (with $r_{1}\in R_{\mathrm{root}1}$, $r_{2}\in R_{\mathrm{root}2}$) used to generate the random access sequences are derived according to Algorithm 2, as detailed in Table 1 and Table 2. Table 1 corresponds to scenarios requiring a maximum of I=64 random access preamble sequences per beam coverage, while Table 2 addresses I=128. Here, the length of the short ZC sequence $N_{ZC}$ is set to 839 to maintain compatibility with the TN random access sequence design [32], resulting in $N=K\cdot N_{ZC}=6712$.

**Figure 4:**
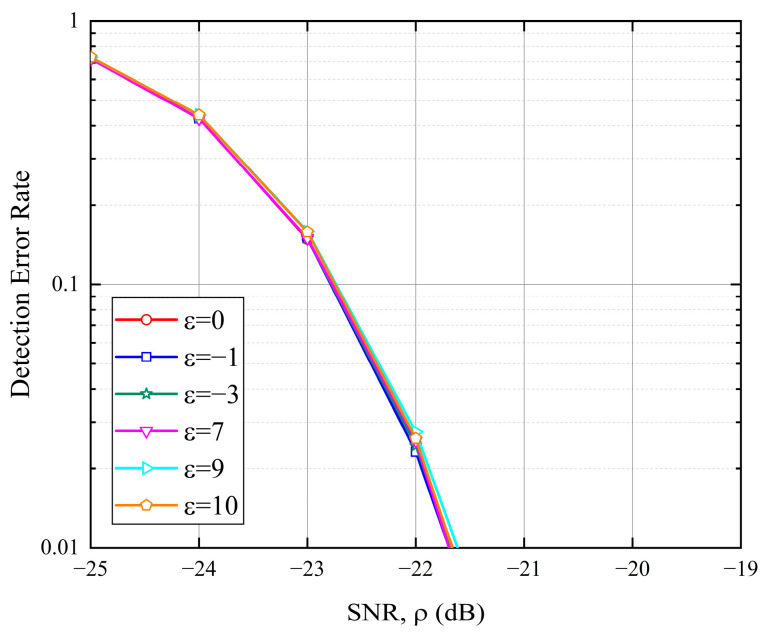
Detection error rate under various integer CFOs with K=8 and $N=K\cdot N_{ZC}=6712$.

During each random access opportunity, the transmitted preamble sequence is randomly selected from a sequence set with I sequences. The actual arrival time is assumed to be randomly (uniformly) distributed. The detection threshold is set to maintain an average false alarm rate of 0.1%. Successful detection is defined as the correct identification of both the presence of the transmitted sequences and their actual arrival timings; otherwise, the detection is considered a failure. As shown in Figure 4, the detection performance in the presence of an integer frequency offset is identical to that without any frequency offset, indicating that integer frequency offsets have no detrimental impact on performance.

**Figure 5:**
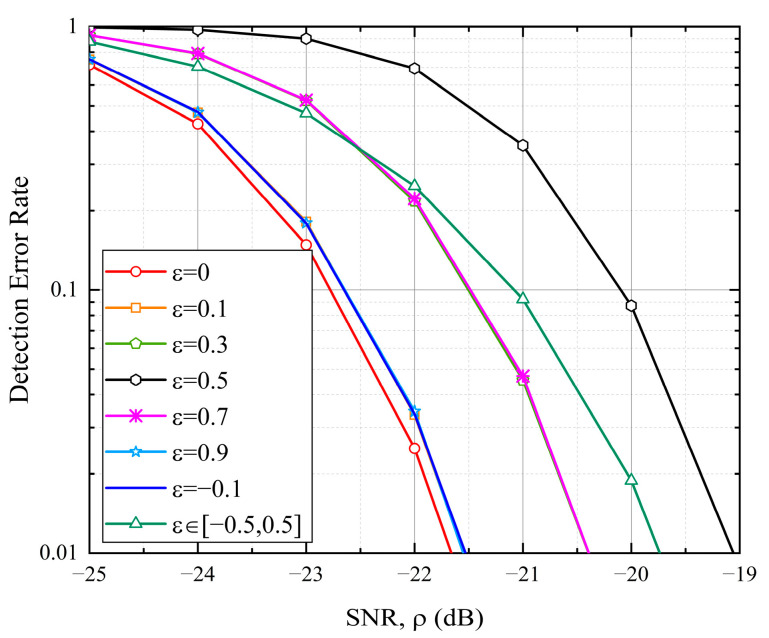
Detection error rate under various non-integer CFOs with K=8 and $N=K\cdot N_{ZC}=6712$. The absolute fractional parts of these CFOs ($\left|\varepsilon_{f}\right|$) range from 0 to 0.5. Specifically, different CFOs can correspond to the same $\left|\varepsilon_{f}\right|$, such as ε=0.1, ε=0.9, and ε=−0.1.

Figure 5 demonstrates the detection performance for random access sequences under various non-integer frequency offsets. It is observed that frequency offsets with identical absolute fractional components ($\left|\varepsilon_{f}\right|$) exhibit equivalent detection performance. For instance, the performance curves for ε=0.1, ε=0.9, and ε=−0.1 (all with $\left|\varepsilon_{f}\right|=0.1$) match perfectly. Similarly, offsets such as ε=0.3, ε=0.7, and ε=−0.3 yield identical results, as shown in Figure 5. The detection performance gradually degrades as $\left|\varepsilon_{f}\right|$ increases (where $0\leq \left|\varepsilon_{f}\right|\leq 0.5$), with the worst performance occurring at $\left|\varepsilon_{f}\right|=0.5$. Compared to $\left|\varepsilon_{f}\right|=0$, there is a limited performance loss of approximately 2.6 dB in the worst-case scenario (i.e., $\left|\varepsilon_{f}\right|=0.5$) to maintain a detection error rate of 1%. The loss will be around 2 dB when $\varepsilon_{f}$ is uniformly distributed in the range of $\left[-0.5,0.5\right]$.

**Figure 6:**
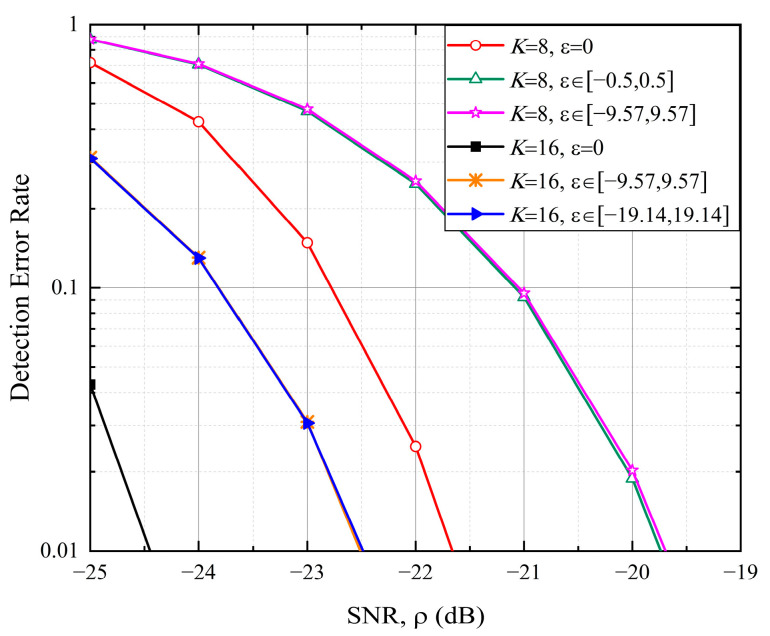
Detection error rate for random access with different length $N=K\cdot N_{ZC}$.

To extend coverage, the length N of the proposed sequence can be increased. Figure 6 presents the detection performance for K=16 and $N=K\cdot N_{ZC}=13,424$. Results demonstrate a performance improvement of approximately 2.8 dB over K=8 within the same normalized CFO range, e.g., $\varepsilon \in \left[-9.57,9.57\right]$. Furthermore, the performance curves for $\varepsilon \in \left[-0.5,0.5\right]$ and $\varepsilon \in \left[-9.57,9.57\right]$ exhibit close alignment for *K* = 8. Similarly, for K=16, the curves for $\varepsilon \in \left[-9.57,9.57\right]$ and $\varepsilon \in \left[-19.14,19.14\right]$ align closely. These results validate the CFO resilience of the proposed random access signal.

.

## 7. Conclusions

This paper proposes a novel design of a random access signal pool to address the scalability challenge in satellite–terrestrial integrated communication systems, where supporting massive connectivity within a single beam is critically required. The proposed solution ensures compatibility with terrestrial network designs while accommodating satellite-specific impairments including wide coverage, large propagation delays and nonignorable device-specific CFO. Key mathematical properties of the sequence roots were analytically derived to enable precise timing estimation under satellite-specific scenario. The accompanying low-complexity detection and timing estimation procedure leverages these properties for robust detection performance. Crucially, the proposed sequences exhibit CFO resilience: integer CFO causes no performance degradation, while fractional CFO induces a maximum loss of merely 2.6 dB in detection probability. Furthermore, an analytical framework was established to guide the practical selection of roots for constructing a large pool of sequences meeting system capacity demands. This framework provides a foundation for designing scalable, interference-resistant preambles tailored to grant-free random access in future satellite systems. The presented signal pool design and analytical results offer a significant reference for preamble standardization and system optimization in next-generation satellite–terrestrial integrated networks, advancing their feasibility and performance.

## Figures and Tables

**Table 1:** Root set selection results when I=64.

Root	Results
$R_{\mathrm{root}1}$	1,5,9,13,17,21,25,29,33,37,41,45,49,53,57,61,65,69,73,77,81,85,89,93,97,101,105,109, 113,117,121,125,129,133,137,141,145,149,153,157,161,165,169,173,177,181,185,189, 193,197,201,205,209,213,217,221,225,229,233,237,241,245,249,253
$R_{\mathrm{root}2}$	3,7,11,15,19,23,27,31,35,39,43,47,51,55,59,63,67,71,75,79,83,87,91,95,99,103,107,111, 115,119,123,127,131,135,139,143,147,151,155,159,163,167,171,175,179,183,187,191, 195,199,203,207,211,215,219,223,227,231,235,239,243,247,251,255

**Table 2:** Root set selection results when I=128.

Root	Results
$R_{\mathrm{root}1}$	1,5,9,13,17,21,25,29,33,37,41,45,49,53,57,61,65,69,73,77,81,85,89,93,97,101,105,109,113, 117,121,125,129,133,137,141,145,149,153,157,161,165,169,173,177,181,185,189,193,197, 201,205,209,213,217,221,225,229,233,237,241,245,249,253,257,261,265,269,273,277,281, 285,289,293,297,301,305,309,313,317,321,325,329,333,337,341,345,349,353,357,361,365, 369,373,377,381,385,389,393,397,401,405,409,413,417,421,425,429,433,437,441,445,449, 453,457,461,465,469,473,477,481,485,489,493,497,501,505,509
$R_{\mathrm{root}2}$	3,7,11,15,19,23,27,31,35,39,43,47,51,55,59,63,67,71,75,79,83,87,91,95,99,103,107,111,115, 119,123,127,131,135,139,143,147,151,155,159,163,167,171,175,179,183,187,191,195,199, 203,207,211,215,219,223,227,231,235,239,243,247,251,255,259,263,267,271,275,279,283, 287,291,295,299,303,307,311,315,319,323,327,331,335,339,343,347,351,355,359,363,367, 371,375,379,383,387,391,395,399,403,407,411,415,419,423,427,431,435,439,443,447,451, 455,459,463,467,471,475,479,483,487,491,495,499,503,507,511

## Data Availability

The original contributions presented in this study are included in the article. Further inquiries can be directed to the corresponding author.
